# Correlation of treatment time to target volume for GammaPod treatments: A simple second calculation

**DOI:** 10.1002/acm2.13524

**Published:** 2022-02-07

**Authors:** Leah Chen, Stewart J. Becker, Sarah Anne McAvoy, Elizabeth M. Nichols, Mariana Guerrero

**Affiliations:** ^1^ Department of Radiation Oncology University of Maryland Baltimore Maryland USA

**Keywords:** GammaPod, second calculation

## Abstract

**Purpose:**

The GammaPod is a novel device for stereotactic breast treatments that employs 25 rotating Co‐60 sources while the patient is continuously translated in three axes to deliver a highly conformal dose to the target. There is no commercial software available for independent second calculations. The purpose of this study is to determine an efficient way to estimate GammaPod treatment times based on target volume and use it as a second calculation for patient‐specific quality assurance.

**Methods:**

Fifty‐nine GammaPod (Xcision Medical Systems, LLC.) breast cancer patient treatments were used as the fitting dataset for this study. Similar to the Curie‐seconds concept in brachytherapy, we considered dose‐rate × time/(prescribed dose) as a function of target volumes. Using a MATLAB (Mathworks, Natick, MA, USA) script, we generated linear (with 95% confidence interval (CI)) and quadratic fits and tested the resulting equations on an additional set of 30 patients.

**Results:**

We found a strong correlation between the dose‐rate × time/(prescribed dose) and patients’ target volumes for both the linear and quadratic models. The linear fit was selected for use and using the polyval function in MATLAB, a 95% CI graph was created to depict the accuracy of the prediction for treatment times. Testing the model on 30 additional patients with target volumes ranging from 20 to 188 cc yielded treatment times from 10 to 25 min that in all cases were within the predicted CI. The average absolute difference between the predicted and actual treatment times was 1.0 min (range 0–3.3 min). The average percent difference was 5.8% (range 0%–18.4%).

**Conclusion:**

This work has resulted in a viable independent calculation for GammaPod treatment times. This method has been implemented as a spreadsheet that is ready for clinical use to predict and verify the accuracy of breast cancer treatment times.

## 1 INTRODUCTION

For most modern radiation treatments, a treatment planning system (TPS) is used to calculate a patient's radiation dose, dose‐rates, and/or treatment times. In conjunction with the TPS, an independent second calculation is regularly used to verify the accuracy of the original dose calculations. The second calculation serves to ensure patient safety and prevents potential gross errors, which is particularly important in new clinical devices. The GammaPod is a novel stereotactic body radiation therapy device that has only recently been cleared for use in 2017.^1^, ^2^, ^3^, ^4^, ^5^, ^6^, ^7^, ^8^ This machine treats breast cancer with highly conformal stereotactic dose distributions delivered with 25 rotating Co‐60 sources collimated to either 25 or 15 mm while the patient is moving. Accurate treatments are ensured by utilizing a unique vacuum cup system that both immobilizes the breast and defines a stereotactic coordinate system with an embedded wire.^9^, ^10^ This allows the GammaPod to spare a larger volume of healthy breast tissue and other organs at risk from radiation exposure. Due to the novelty of the GammaPod, there is no commercially available independent second calculation at this time. The purpose of this research is to create an efficient, independent calculation for verification of GammaPod patient treatment times. This technical note explains the process to develop an equation to estimate treatment time based on target volume as well as a 95% confidence interval (CI). We also discuss the verification of its accuracy as well as analysis of the equation's clinical implementation.

## 2 METHODS

**FIGURE 1:**
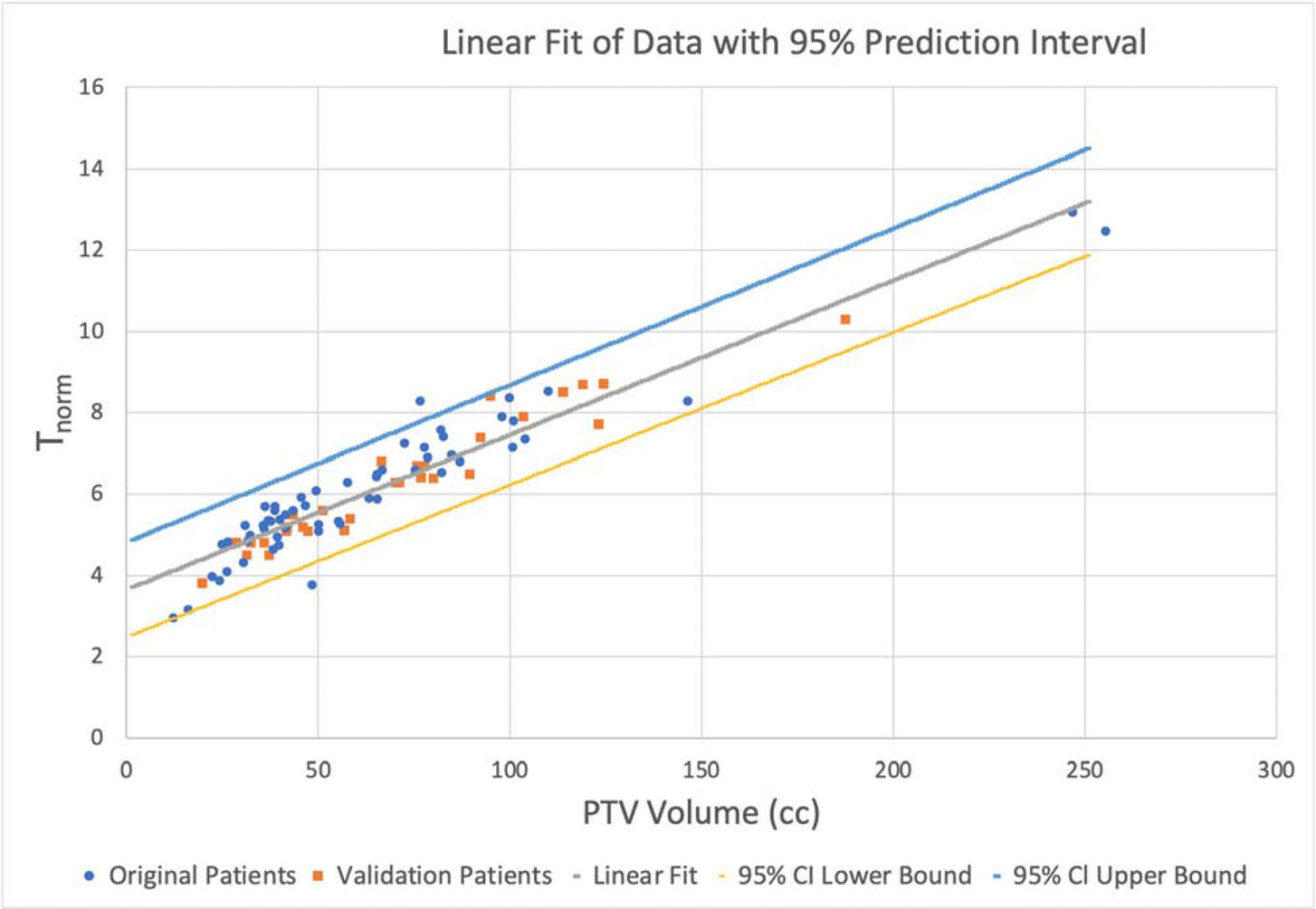
Linear fit of *T*_NORM_ (dose‐rate × time/(prescribed dose)) versus PTV target volume (cc) calculation with the 95% confidence interval (CI). Both fitting and testing data are plotted

Fifty‐nine breast cancer patient plans were included in the study. Thirty‐four patients with Planning Target Volumes (PTV) from 12 to 98 cc, received a GammaPod boost of 8 Gy to the lumpectomy cavity with a Clinical Target Volume (CTV) margin of 0.5 cm and a PTV margin of 0.3 cm based on previous studies combined with standard external beam treatment.^9^ Twenty‐five patients (PTV volumes from 41.4 to 391 cc) were treated exclusively using the GammaPod for accelerated partial breast irradiation (APBI) to doses of 30–40 Gy in five fractions to the lumpectomy cavity with a 1 cm CTV margin and 0.3 cm PTV margin. We used the patients’ plan reports to collect information on their individual target volumes, treatment times, prescribed dose to 95% of the target volume, and dose‐rates. Since treatment times depend on dose‐rate and dose, we normalized the treatment time by multiplying by the dose‐rate and dividing by prescription dose. This is similar to the concept of Curie‐seconds in brachytherapy, which derives from mgRa‐hours, the original quantity used to characterize radium implants.^11^ This normalized treatment time (*T*_NORM_) is a unitless quantity that correlates strongly with treatment volume. We considered a linear and a quadratic fit to the data. To avoid data crowding and random fluctuations, the PTV target volumes and times of patients with five‐fraction treatments were averaged. Then, we filtered the data of outliers by limiting our PTV target volume range to between 12 and 256 cc (the treatments for the patient with the largest target volume were outliers and not from primary breast cancer but rather an unusual palliative case). Figure 1 shows the 58 data points used for fitting and the linear fit with the 95% CI. To determine if a linear or quadratic model was more suited to fit the data, we used the polyfit function in MATLAB (Mathworks, Natick, MA, USA). The quality of the fit was evaluated using the *R* ^2^ value calculated by MATLAB.

## 3 RESULTS

The difference between goodness of fit for the quadratic model and the linear model was negligible (*R* ^2 ^= 0.91 for the quadratic and *R* ^2 ^= 0.895 for the linear model) so for simplicity, we decided to use the linear model, generating the equation

(1) $$T_{\mathrm{NORM}}=0.03802\;cc^{-1}\times \mathrm{PT}V_{\mathrm{Vol}}\left(\mathrm{cc}\right)+3.689$$

Using the polyval function in MATLAB again, we constructed a model for the 95% CI of this linear fitting, where 95% of all data points fell between the upper and lower bounds of the graph. Figure 1 shows the linear fit with the CI.

The function of the normalized treatment time CI is:

(2) $$\begin{array}{ccc} \text{Lower}\;\text{bound}:T_{\mathrm{NORM}} & = & 0.03747cc^{-1}\\ & & \times \;\mathrm{PT}V_{\mathrm{Vol}}\left(\mathrm{cc}\right)+2.519 \end{array}$$

(3) $$\begin{array}{ccc} \text{Upper}\;\text{bound}:T_{\mathrm{NORM}} & = & 0.03857cc^{-1}\\ & & \times \;\mathrm{PT}V_{\mathrm{Vol}}\left(\mathrm{cc}\right)+4.86 \end{array}$$

Treatment time (*T*_TREAT_) and its CI can be estimated by solving the equation for the treatment time variable: *T*_TREAT_ = *T*_NORM_ × dose/dose‐rate.

We utilized Equation (1) on a validation set of 30 additional patients to predict treatment times based on their PTV volume, prescribed dose, and dose‐rate and then compared those times with the actual treatment times.

In order to create a system for clinical use, we used Excel (Microsoft) to generate a spreadsheet that estimates treatment times based on the user input of patient prescribed dose, PTV volume, and dose‐rate. Using Equations (1)–(3) in the spreadsheet, we predicted the treatment time as well as the upper and lower bounds to which the actual treatment time should fall. This way, the GammaPod TPS calculated treatment time can be cross‐referenced with the spreadsheet predicted treatment time.

The actual treatment times and estimated times for the 30 validation set patients were compared. The target volumes in the 30 patients ranged from 20 to 188 cc, and the treatment times ranged from 10 to 25 min. Absolute values of the percent time differences averaged 5.8% (range 0.3%–18.4%) and absolute values of time differences in minutes averaged 1.0 min (range 0–3.3 min). All of the actual treatment times were within the 95% CI from Equations (2) and (3). The data for the 30 patients is also plotted in Figure 1 with different symbols.

The patient data used for fitting are patients that have a single dose prescribed to 95% of the PTV. Among the 30 test patients, there are nine from a protocol that has two dose levels: one dose prescribed to 95% of CTV (typically 8 Gy) and another dose (typically 6 Gy) to 95% of PTV. For these patients, the high dose level and the CTV volume were used for testing our model. The average difference between the actual and estimated times for the two‐dose‐level patients was 6.9% (range 3.2%–13.3%) versus 5.3% (range 0.3%–18.4%) for the single‐dose patients, which is not statistically significant (*p*‐value = 0.37). This suggests that our model can be used for the two‐dose‐level patients using the larger dose to the CTV volume, although a separate analysis may be warranted for those patients.

## 4 DISCUSSION

Currently, there is no commercially available independent second calculation for the GammaPod TPS. One of the institutions using the GammaPod clinically is using an in‐house Monte Carlo code as a second calculation. While a Monte Carlo‐based second calculation could be very powerful, it is not readily available for all users. Our work is intended to fill the gap in lack of second calculation with a simple method that can be easily implemented in any clinic that has Excel (Microsoft) or any other spreadsheet software available. In the current analysis, there are only a few data points in the fitting data above 100 cc or so, and one of them is near the lower CI limit. However, the six patients in the validation set that had target volumes near or above 100 cc fall well within the CI. As more data with larger target volumes become available, we will evaluate if a tweak of the fitting is necessary to better describe larger volumes.

It is worth noticing that while the width of the CI in the time × dose‐rate/dose is approximately 2.4 for all cases, this translates to a larger uncertainty in treatment time for larger doses and lower dose‐rates, which can be of the order of a few minutes. It is not straightforward to evaluate the uncertainty in the dose given by the CI because of the nature of the treatment. Different areas of the target are irradiated at different times, so the CI uncertainty is translated into a complex combination of target volume interval and dose interval rather than the overall dose.

Our estimation of treatment time relies on the correlation between PTV target volume and treatment time. Our data show that for a given PTV target volume there can be a significant range of treatment times, due to factors like the location of the lumpectomy cavity, collimator diameter, and breast size that are not captured in our simple approach. This range of treatment times for a given PTV volume is reflected in a rather large CI in estimated treatment times. In order to consider additional factors beyond the target volume, given the complexity of the treatment, a machine learning type of approach may be needed. In our study, most cases had the same dose to PTV and CTV. For cases where the CTV is prescribed to a higher dose than the PTV, the model was tested using the larger dose and the CTV volume with comparable results to the one‐dose‐level case, although an independent analysis may be warranted with a larger number of two‐dose‐level patients.

In our field, we typically keep the dose uncertainty in the treatments to less than 5% as is recommended in several AAPM Task Group reports like TG40^12^ and TG114.^13^ Our proposed approach may not meet this standard since the average percent difference between the estimated and actual treatment times was 5.8% (range 0%–18.4%), which can potentially translate to dose differences larger than 5%. However, it is an important first step, particularly given that this is a new device with limited clinical use and potentially undiscovered errors and that may otherwise be used without a second calculation at all. As an example, we recently had a patient case where there was a glitch in the optimization software. For that patient, one fraction had the PTV volume as 106.5 cc, the prescription dose was 6 Gy to 95% of the PTV, and the dose‐rate was 2.21 Gy/min. Our calculation predicted 21.0 min (CI 17.7–24.3 min). The first optimization of the plan gave a treatment time of 29 min, well beyond our upper bound. The longer plan ended up being non‐deliverable by the control system. The reasons for this problem are still under investigation but given the novelty of this software, it is conceivable that other glitches may be present, and our calculation can help filter at least some of them. After the faulty plan was reoptimized, the reoptimized plan had a 19‐min treatment time (consistent with our calculation) and was delivered with no problems. Our method can provide a sanity check and potentially avoid large errors in patient treatments. Our method is similar to the Curie‐seconds estimate in interstitial brachytherapy, where treatment times multiplied by source activity per unit dose can be used as a check for patient treatment time, although details about the number of catheters and patient geometry are not included.

## 5 SUMMARY AND CONCLUSION

We successfully extracted and organized breast cancer patient data to create an independent calculation for GammaPod treatment times. Using a linear fit of data, the treatment time was adjusted for dose and dose‐rate and was correlated with PTV target volume. The equation and the 95% CI were tested in 30 new patients with a wide range of target volumes. The test patients’ treatment times given by the TPS were always within the expected CI, confirming that our generated calculation is now ready for clinical use.

## CONFLICT OF INTEREST

None.

## AUTHOR CONTRIBUTIONS

Leah Chen organized and analyzed the data, and drafted the article (all under guidance). Stewart J. Becker helped in designing the IRB protocol for data collection, critically reviewing the project design, and the manuscript writing. Sarah Anne McAvoy contributed with patients’ data acquisition and critical review of the work and manuscript writing. Elizabeth M. Nichols contributed with patients’ data acquisition and critical review of the work, and also helped in designing the IRB protocol for data collection. Mariana Guerrero did initial project design, provided direct guidance for Ms. Chen, and performed initial manuscript review. All authors gave final approval of the manuscript.
